# Effects of a β-Glucan-Rich Blend of Medicinal Mushrooms and Botanicals on Innate Immune Cell Activation and Function Are Enhanced by a Very Low Dose of Bovine Colostrum Peptides

**DOI:** 10.3390/molecules29122787

**Published:** 2024-06-12

**Authors:** Gitte S. Jensen, Dina Cruickshank, Debby E. Hamilton

**Affiliations:** 1 NIS Labs, 1437 Esplanade, Klamath Falls, OR 97601, USA; 2 NIS Labs, 807 St. George St., Port Dover, ON N0A 1N0, Canada; dina@nislabs.com; 3 Researched Nutritionals, P.O. Box 224, Los Olivos, CA 93441, USA; drdebby@holisticpediatric.com

**Keywords:** β-glucans, CD69, IL-1β, IL-1ra, IL-6, IL-10, K562, TNF-α

## Abstract

Nutraceutical immune support offers potential for designing blends with complementary mechanisms of action for robust support of innate immune alertness. We documented enhanced immune activation when bovine colostrum peptides (BC-Pep) were added to an immune blend (IB) containing β-glucans from yeast, shiitake, maitake, and botanical non-β-glucan polysaccharides. Human peripheral blood mononuclear cells (PBMCs) were cultured with IB, BC-Pep, and IB + BC-Pep for 20 h, whereafter expression of the activation marker CD69 was evaluated on NK cells, NKT cells, and T cells. Cytokine levels were tested in culture supernatants. PBMCs were co-cultured with K562 target cells to evaluate T cell-mediated cytotoxicity. IB + BC-Pep triggered highly significant increases in IL-1β, IL-6, and TNF-α, above that of cultures treated with matching doses of either IB or BC-Pep. NK cell and T cell activation was increased by IB + BC-Pep, reaching levels of CD69 expression several fold higher than either BC-Pep or IB alone. IB + BC-Pep significantly increased T cell-mediated cytotoxic killing of K562 target cells. This synergistic effect suggests unique amplification of signal transduction of NK cells and T cells due to modulation of IB-induced signaling pathways by BC-Pep and is of interest for further pre-clinical and clinical testing of immune defense activity against virally infected and transformed cells.

## 1. Introduction

Nutraceutical support of the immune system is an important part of managing inflammatory conditions and maintaining good health. There is an increased focus on supporting a healthy immune defense activity through immunomodulation [1,2], which includes both the initial acute pro-inflammatory response and support of anti-inflammatory mechanisms of returning to baseline.

Botanical and fungal food sources contain many types of non-starchy polysaccharides with immunogenic properties. Among these, β-glucans are glucose polymers found in the cell walls of plants and fungi and serve as a major recognition mechanism and activation pathway in antifungal immunity. There is considerable structural diversity between β-glucans from different kingdoms of living organisms [3] and a possibility for higher animals to react in different ways to plant-derived versus fungal-derived β-glucans, which suggests that a blend of plant-based and mushroom-based β-glucans may be advantageous. Examples of non-β-glucan polysaccharides come from the root of the medicinal herb astragalus [4], which has profound effects on the maturation state of macrophages and dendritic cells [5], and arabinogalactan, a form of hemicellulose with immune-modulating properties. 

Our immune system responds to potentially harmful substances rapidly and communicates system-wide by means of cytokine secretion. Immune cells express pattern recognition receptors (PRRs) for the recognition of molecular patterns associated with different microbial lifeforms [6,7]. Immune cell PRRs include toll-like receptors (TLRs), C-type lectin receptors (CLRs), including the Dectin-1 and Dectin-2 receptors involved in anti-fungal immune protection [8,9,10], nucleotide-binding oligomerization domain-like receptors (NLR) [11,12], and retinoic acid-inducible gene-I-like receptors [13]. Whereas NLRs and RLRs function as intracellular sensors of pathogen-associated molecular patterns, TLRs and CLRs are expressed on the cell surface and are relevant for the initial recognition of the nutraceutical botanical and fungal immune modulators described here. These receptors are well characterized for the recognition of bacterial and fungal antigens, including β-glucans. CLRs also recognize patterns associated with plant-based polysaccharides such as arabinogalactans, arabinoxylans, and alpha-mannans [14,15,16,17]. Members of the family of toll-like receptors are able to recognize bacterial, fungal, and viral epitopes, as well as botanical-derived polysaccharides [18]. Immune cell activation via both the TLR-type receptors and the Dectin-1 receptor leads to NFkB activation; however, the different types of receptors do so via different signaling pathways [19]. The engagement of PRRs on antigen-presenting immune cells such as macrophages, dendritic cells, and natural killer (NK) cells leads to an elevated state of alertness [20]. The simultaneous stimulation of immune cells via multiple PRRs has synergistic effects [21].

Shifting the focus from botanical to animal-based immunogenic compounds, our food is scarce in some of the most robust immune-protective substances in the animal kingdom. In nature, immunogenic peptides play pivotal roles in protecting the young by transmission of immune defense capacity from the mature and educated immune system of the mother. Newborn mammals receive a broad spectrum of immunogenic proteins, peptides, glycolipids, and hormonal factors through feeding on colostrum, which is the first milk the mother produces after giving birth [22]. Antibacterial peptides are also present in egg yolk as a method to transfer immunity from the mother bird to the developing embryo in the egg [23]. These immunogenic peptides are not species-specific, and peptides from one species, whether mammalian or avian, can protect another species, and peptides produced by one individual can be absorbed orally by another individual. This has led to commercialization of nutritional supplements from sustainable and non-invasive sources of immunogenic peptides such as bovine colostrum. Immunogenic low molecular weight peptides can be extracted and concentrated through ultrafiltration, and the safety of ultrafilrates when consumed by other mammals has been documented in a rodent model [24]. 

Colostrum is rich in peptides, including proline-rich peptides, transfer factors, and growth factors [25]. Proline-rich peptides contain 25% proline amino acids and have unique immune-regulating properties, specifically regarding Th1/Th2 cytokine induction [26] and cytotoxic T cells [27]. Transfer factors [28] include antigen-recognizing molecules with specific roles in T cell-mediated immune responses [29], specifically for the cytotoxic response where some types of transfer factors tag transformed and virally infected cells for destruction, aiding cytotoxic T cells to recognize and kill virally infected cells in the body [30].

Bovine colostrum from healthy cows can transfer immune protection against potential pathogens that the cow has encountered to other mammals and to birds [31,32]. Bovine colostrum ultrafiltrates have been studied in vitro and in rodent models for bacterial and viral upper respiratory tract infections. Our team has previously shown that these ultrafiltrates showed rapid effects where phagocytic activity was enhanced within minutes in vitro, and nasopharyngeal treatment with the ultrafiltrate shortly before viral or bacterial infection significantly increased survival and increased microbial clearance in a rodent model, with reduced bacterial and viral loads seen in the lungs at 24 h [33]. We also documented in a clinical cross-over trial that when consumed by healthy humans, cellular immune responsiveness was significantly increased within 2 h after consumption [34].

The activities of colostrum peptides have the potential for synergy with other natural products with immune-activating properties, providing an opportunity to enhance nutraceutical immune support through a complex oral formulation. Examples include β-glucans as anti-infectious agents [35]. We have documented through clinical trials that yeast-based β-glucan containing consumable products provide support of mucosal immunity [36] and provide rapid immune-modulating effects in vitro including immune surveillance and changes to cytokine levels [37,38]. We have found that medicinal mushrooms prime immune cells through pattern-recognizing receptors and directly trigger increased NK and T cell activation and cytokine production [39,40]. 

A nutraceutical multi-ingredient nutritional supplement was formulated to strengthen the immune system. The product contains bovine colostrum low molecular weight peptides (BC-Pep), along with immune-supportive mushrooms, herbs, zinc, and selenium. Previous research on the nutraceutical product was performed by Nicholson’s team and showed an increase in natural killer cell activity in patients whose blood samples were in the lower reference range for natural killer cell function [41]. The research reported here was designed to evaluate the contributions from the mushroom and botanical immune blend (IB) versus the BC-Pep fraction, and document potential synergistic effects by the botanical components versus the colostrum-based ingredients on human immune cells in vitro. 

## 2. Results

### 2.1. Production of Cytokines

**Figure 1:**
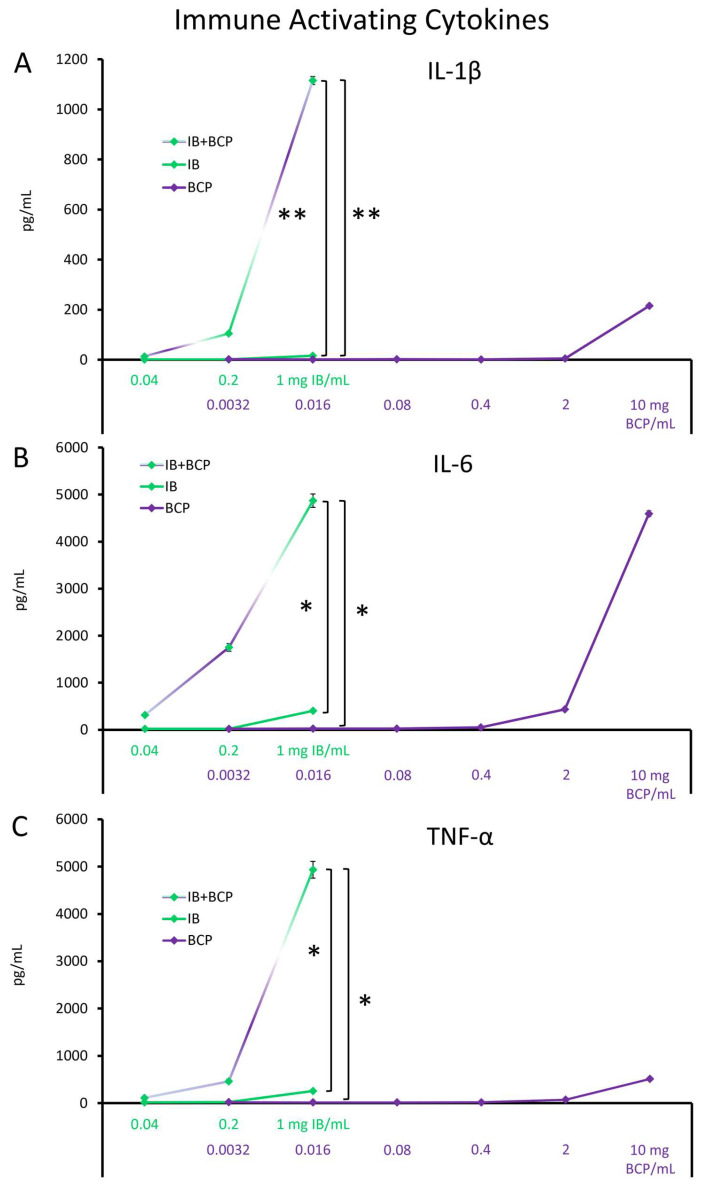
Induction of immune-activating cytokines: (**A**) IL-1β, (**B**) IL-6, and (**C**) TNF-α. The average ± standard deviation is shown, where the immune blend (IB) and the bovine colostrum low molecular weight peptides (BC-Pep) are compared to the immune blend with BC-Pep (IB + BC-Pep), tested across a dose range of 0.04–1 mg/mL cell culture. As a reference, BC-Pep was tested across a dose range of 0.0032–10 mg/mL cell culture. The graphs are aligned to show the effect of IB + BC-Pep to matching doses of IB and BC-Pep. Human PBMCs were cultured with test products in vitro for 20 h. The culture supernatants were tested for immune-activating pro-inflammatory cytokine levels. The statistical significance when comparing the test products is indicated with * for *p* < 0.05, and ** for *p* < 0.01.

The culture supernatants from the PBMC cultures described above were tested for levels of immune-activating pro-inflammatory cytokines (Figure 1). BC-Pep at the highest dose of 10 mg/mL triggered immune-activating events leading to induction of interleukin-1-beta (IL-1β, Figure 1A), interleukin-6 (IL-6, Figure 1B), and tumor necrosis factor-alpha (TNF-α, Figure 1C). However, the immune-activating effects of BC-Pep alone declined at lower doses and were undetectable at the dose of 0.016 mg BC-Pep/mL cell culture. The IB without BC-Pep triggered increases of these cytokines at the dose of 1 mg IB/mL cell culture. The blend of IB + BC-Pep at that dose contained 0.016 BC-Pep/mL, i.e., the dose where effects of BC-Pep were undetectable. At that dose, the IB + BC-Pep blend triggered many-fold higher levels of all three cytokines than IB alone. For IL-1β, the amplified induction of IL-1β by IB + BC-Pep above that of BC-Pep or IB alone was highly significant (*p* < 0.01, Figure 1A). For both IL-6 and TNF-α, the induction by IB + BC-Pep over BC-Pep or IB alone was statistically significant (*p* < 0.05, Figure 1B,C). 

**Figure 2:**
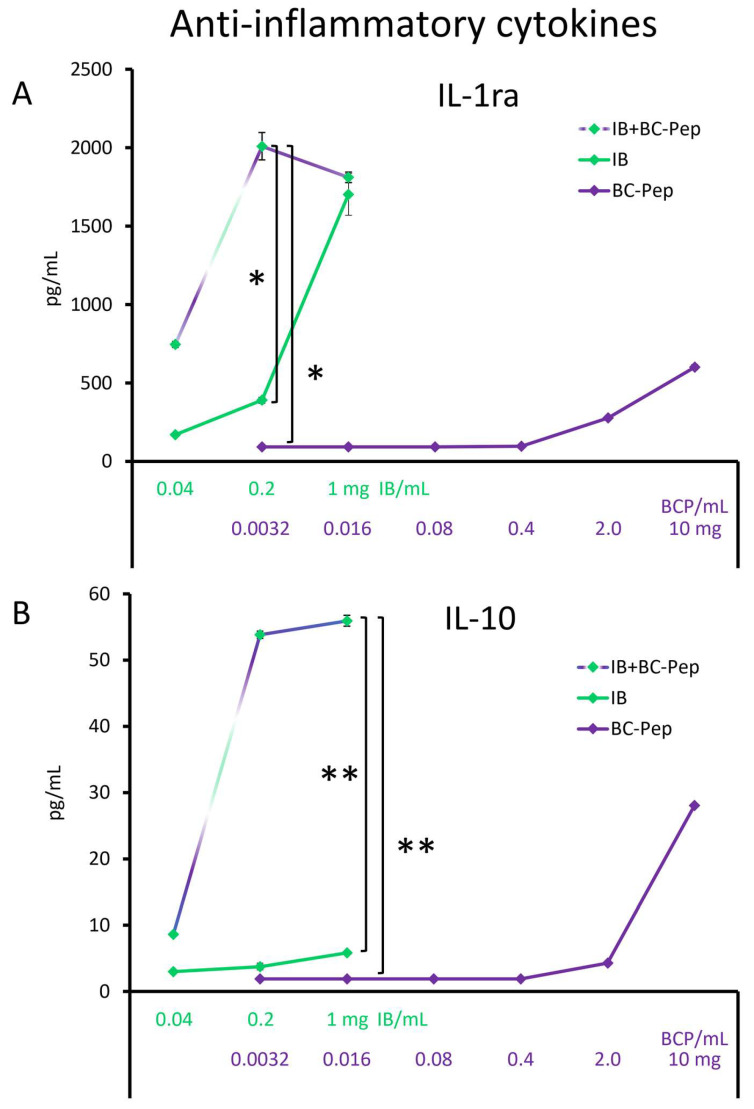
Induction of anti-inflammatory cytokines: (**A**) interleukin-1 receptor antagonist (IL-1ra) and (**B**) interleukin-10 (IL-10). The average ± standard deviation is shown, where the immune blend (IB) and the bovine colostrum low molecular weight peptides (BC-Pep) are compared to the immune blend with IB + BC-Pep, tested across a dose range of 0.04–1 mg/mL cell culture. As a reference, BC-Pep was tested across a dose range of 0.0032–10 mg/mL cell culture. The graphs are aligned to show the effect of IB + BC-Pep to matching doses of IB and BC-Pep. Human PBMCs were cultured with test products in vitro for 20 h. The culture supernatants were tested for immune-activating pro-inflammatory cytokine levels. The statistical significance when comparing the test products is indicated with * for *p* < 0.05 and ** for *p* < 0.01.

The culture supernatants from the PBMC cultures described above were tested for levels of anti-inflammatory cytokines (Figure 2). BC-Pep at the highest dose of 10 mg/mL triggered anti-inflammatory effects involving induction of interleukin-1 receptor antagonist (IL-1ra, Figure 2A) and interleukin-10 (IL-10, Figure 2B). However, the effects of BC-Pep alone declined at lower doses and were negligible at the dose of 0.016 mg BC-Pep/mL cell culture. For IL-10, the induction by IB + BC-Pep over BC-Pep or IB alone was highly significant at the dose of 1 mg IB/mL cell culture (*p* < 0.01, Figure 2B). 

Both the IB with and without BC-Pep triggered similar increases in IL-1ra levels at the dose of 1 mg IB/mL cell culture (Figure 2A). However, at a dose of 0.2 mg IB/mL, there was a robust and statistically significant difference between IB versus IB + BC-Pep, where IB + BC-Pep inducing significantly higher levels of IL-1ra than IB or BC-Pep alone (*p* < 0.05). 

### 2.2. Immune Cell Activation

Human peripheral blood mononuclear cells were used to evaluate the direct immune-activating properties of IB with and without BC-Pep, compared to BC-Pep alone. The cells were treated with test products for 20 h, and the induction of the early activation marker CD69 was evaluated on CD3− CD56+ NK cells, CD3+ CD56+ NKT cells, and CD3+ CD56− T cells. This was compared to CD69 induction by BC-Pep alone, tested at the matching dose (Table 1). The CD69 induction on NK cells treated with the IB + BC-Pep blend was four times higher than that on NK cells treated with IB alone (not significant). The increased CD69 expression on NKT and T cells was significantly higher than on cells treated only by BC-Pep (*p* < 0.05). The increased expression of CD69 in cultures treated with IB + BC-Pep was higher than on cells treated only with IB and reached statistical significance for T cells (*p* < 0.05) and a high level of statistical significance on NKT cells (*p* < 0.01).

### 2.3. Immune Cell Recognition of Transformed Target Cells

**Figure 3:**
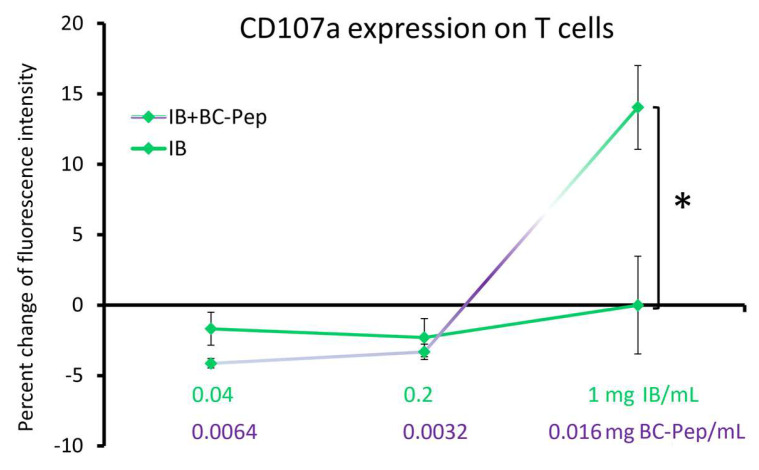
Cell surface expression of CD107a as a measure of degranulation during cytotoxic activity towards K562 target cells in PBMC/K562 co-cultures. Human PBMCs were co-cultured with the MHC Class I deficient target cell K562, untreated or stimulated in vitro for 6 h with IL-2 (100 IU/mL), or each of the two test products (IB and IB + BC-Pep). The dose of the immune blend (IB) was 1 mg/mL and the dose of the bovine colostrum low molecular weight peptides (BC-Pep) was 16 µg/mL in IB + BC-Pep. The cells were stained and analyzed by flow cytometry for expression of CD107a on CD3+ CD56− T cells. The statistical significance when comparing the test products is indicated with * for *p* < 0.05.

Peripheral blood mononuclear cells (PBMC) were co-cultured with the MHC class I deficient cell line K562 to allow cytotoxic cells to recognize and kill the K562 target cells through cell–cell contact, degranulation, and secretion of perforin and granzyme from NK and T cells, thereby killing the transformed target cells. During this process, the CD107a marker is transiently expressed on the cell surface of cytotoxic NK and T cells. In PBMC/K562 co-cultures treated with IB + BC-Pep, an increased CD107a expression was seen specifically on the T cell population, suggesting that IB + BC-Pep enhanced cytotoxic activity of cells within the T lymphocyte population (Figure 3). For the 1 mg IB/mL cell culture, the increased CD107a expression on T cells was statistically significant for IB + BC-Pep when comparing to cells treated with IB alone (*p* < 0.05). 

## 3. Discussion

This work was conducted to document the effects of a botanical and fungal immune blend (IB) on immune cell activation in the absence versus presence of bovine colostrum low molecular weight peptides (BC-Pep). The data reported here have demonstrated that the addition of very low doses of BC-Pep significantly enhanced immune cell activation and recognition of transformed target cells by the nutraceutical immune blend (IB). The testing was performed at doses where the IB provided a mild or moderate activation of immune cells, as measured by expression of the early activation antigen CD69, and a low level of cytokine production. BC-Pep was tested at the low dose of 16 µg/mL, where the BC-Pep fraction alone had an almost negligible effects on immune cell activation and cytokine production. When IB and BC-Pep were blended, a robust enhancement was seen for cytokine production, CD69 expression on NK cells, and of T cell-mediated cytotoxic killing of K562 target cells as a model for a virally infected or malignantly transformed cell. The blend of IB and BC-Pep showed over 1000-fold increases in the production of the 3 immune-activating pro-inflammatory cytokines, IL-1β, IL-6, and TNF-α, compared to either ingredient alone. At the same time, there was an increased production of the 2 anti-inflammatory cytokines IL-1ra and IL-10, suggesting anti-inflammatory support during return to homeostasis. The increased cytotoxic activity when BC-Pep was blended with IB was specifically seen for T lymphocytes, and it was not as apparent for NK cells in the same PBMC cultures. The increased expression of CD69 on NK cells when treated with IB + BC-Pep reached a high level of statistical significance when compared to NK cells treated with either BC-Pep or IB alone (*p* < 0.001). This demonstrates a higher alertness of the activated NK cells as part of the innate immune defense. This increase suggests that the NK cells are also more active, as a direct and highly significant correlation between CD69 levels and NK cell activity was demonstrated by Clausen et al. (2003) in a study involving 14 breast cancer patients tested repeatedly during chemotherapy [42]. 

The underlying mechanisms of enhanced immune cell activation by the IB when presented to immune cells in the presence of very low doses of BC-Pep are unknown. The robust enhancement of multiple innate immune defense activities may suggest regulating effects, likely due to the high content of proline-rich peptides. Proline-rich domains are known for their unique properties in signal transduction, including direct effects, as well as modification of receptor-ligand interactions [43]. This may include competitive inhibition of receptors engaged by natural ligands, or induction of conformational changes of cell membrane receptors during low-affinity, brief-duration molecular engagements. The proline-rich peptides in colostrum from different mammalian species show similar structures [44], and include a heterogenous mixture of over 30 peptides, mainly derived from casein [45], known to modulate cytokine production at doses starting at 5 mg/mL [46]. We confirmed this cytokine induction at similar doses, which in our hands became undetectable at 1 mg/mL and lower doses; however, these lower doses were capable of robustly enhancing the immune-activating effects of the botanical and fungal-based IB. 

The robust magnification of innate immune defense activity by a very low dose of colostrum peptides is puzzling and may have broad therapeutic implications in β-glucan-related immunotherapies. A simple hypothesis may suggest usage of different cell surface receptors by compounds in IB versus BC-Pep, which alone does not produce a detectable response, but when triggered simultaneously at low doses produces an enhanced immune response. This can be approached by similar cell culture experiments, where neutralizing antibodies are added singly and in combinations, directed towards the Dectin-1, Dectin-2, TLR-2, and TLR-4 receptors. The ability of very low doses of BC-Pep to enhance the immune activating and modulating effects of the β-glucan-rich nutraceutical blend IB may in part be due to engagement of different categories of cell surface receptors on different cell types with the peripheral blood mononuclear cell (PBMC) fraction. Therefore, the cytokine responses in the absence and presence of neutralizing antibodies should also be tested on cultures of isolated monocytes, NK cells, and T cells. The comparison of responses in PBMC cultures to isolated cell types may point to the roles of each cell type in the collaborative efforts in PBMC cultures. This cascade of events may be partially responsible for the robustly enhanced cytokine production and the increased cytotoxicity in cultures co-stimulated with both BC-Pep and IB. However, there are other possible explanations, including epigenetic re-programming by compounds from both he botanical sources and the low molecular weight colostrum peptides, and further research is urgently needed. The preliminary in vitro data presented here has triggered many questions, including whether the induction of synergy is related to specific peptides or whether the strong effect depends on the complex blend of peptides naturally present in colostrum. It is also imperative to gain more detailed understanding of whether this effect may be seen in context of a single source of either botanical or fungal non-starch polysaccharides, or whether the complexity of polysaccharides from fungi and botanicals is a critical component of the response.

## 4. Materials and Methods

### 4.1. Reagents

The following buffers and reagents were obtained from Sigma-Aldrich (St. Louis, MO, USA): Phosphate-buffered saline (PBS), RPMI-1640 culture medium, and Lympholyte-Poly density gradient. The fluorescence-labelled monoclonal antibodies CD69-FITC (clone L78), CD107a-FITC (cat 555800), CD56-PE (clone My31) and CD3-PerCP (clone SK7) and Monensin (BD GolgiStop) were obtained from BD Biosciences (San Jose, CA, USA). Sodium azide (NaN3) was obtained from LabChem Inc. (Pittsburgh, PA, USA). 

### 4.2. Nutraceutical Immune Blend and Bovine Colostrum Peptides

A nutraceutical immune blend of herbs, mushroom β-glucans, and minerals, Transfer Factor Multi-Immune^®^ (Researched Nutritionals, Los Olivos, CA, USA), was studied to document its immune activating properties. The commercial blend contains a small dose of bovine colostrum low molecular weight peptides (BC-Pep, 1.5% per weight). The immune blend (IB) without the bovine colostrum peptides was tested alongside the commercial blend containing BC-Pep (IB + BC-Pep). IB contains zinc, selenium, and β-glucans from yeast and the medicinal mushrooms shiitake and maitake, as well as the non-β-glucan botanical polysaccharides arabinogalactan, astragalus. It also contains antioxidant-rich extracts from green tea and pomegranate. The goal was to explore possible synergistic effects of IB versus BC-Pep, to see whether the blend of these fractions would have simple additive effects or could produce highly potent synergistic effects. Therefore, it was important first to identify a dose range where the immune-activating effects of each fraction were recognizable but minimal. For both IB and BCP, the dose where only minimal or no effects on cytokine production in PBMC cultures was 1 mg/mL cell culture. Then the two fractions and the blend thereof were compared in various immune assays, where the BC-Pep was tested at doses that matched its dose in the commercial product.

### 4.3. Purification of Peripheral Blood Mononuclear Cells 

Peripheral blood mononuclear cells (PBMCs) were obtained from healthy human volunteers. The blood donors were between the ages of 20 and 60 years and donated blood with informed consent, as approved by the Sky Lakes Medical Center Institutional Review Board (FWA 2603). Freshly drawn peripheral venous blood samples in sodium heparin were layered onto Lympholyte-Poly (Cedarlane Labs, Burlington, NC, USA) and centrifuged for 35 min at 360× *g*. The upper, PBMC-rich interface was harvested using sterile transfer pipettes and washed twice with 10 mL PBS without calcium or magnesium by centrifugation at 400× *g* for 10 min. Cell counting was performed in a hemocytometer using Trypan Blue exclusion to assure 100% cell viability. The PBMC fraction contains the natural killer (NK) cell population and was used for testing of NK cell cytotoxic activity as well as expression of CD69 and CD25 activation markers. Culture supernatants were tested for cytokine production.

### 4.4. Production of Cytokines, Anti-Viral Peptides, and Growth Factors

Supernatants were harvested from the PBMC cultures and frozen until used for cytokine testing. The levels of 9 cytokines, chemokines, and growth factors were analyzed. The markers IL-1β, IL-1ra, IL-6, IL-10, IFN-γ, and TNF-α were quantified using Bio-Plex protein arrays (Bio-Rad Laboratories Inc., Hercules CA, USA) and utilizing xMAP technology (Luminex, Austin, TX, USA). 

### 4.5. Immune Cell Activation

Freshly isolated PBMCs were distributed in sterile U-bottom 96-well culture plates (NUNC, Denmark) at a concentration of 0.2 × 10^6^ PBMCs in 0.2 mL cell culture and treated with test products, where each condition and dose of test product was tested in triplicate. The experiment was performed three times using cells from three different healthy donors. Untreated PBMC cultures were performed in triplicate and served as negative controls. PMC cultures treated with IL-2 (100 IU/mL) and LPS (10 ng/mL) served as positive controls for CD69 expression and cytokine induction. For activation of natural killer (NK) cells, T cells, non-NK non-T cells, and monocytes, the incubation time was 20 h. Cells were transferred to V-bottom 96-well plates (NUNC Denmark) and washed in PBS containing 1% bovine serum albumin and 0.02% sodium azide. Monoclonal antibodies were added in previously established optimal quantities (CD3-PerCP, CD56-PE, and CD69-FITC: 6.5 μL/sample) and incubated in the dark at room temperature for 10 min. The cells were washed twice with protein-free PBS containing 0.02% azide and then resuspended in 0.05 mL PBS with 0.02% azide and transferred to 0.5 mL polystyrene round-bottom 96-well plates containing 0.3 mL of 1% formalin. Samples were stored in the dark and acquired by flow cytometry within 3 h using an acoustic aligning Attune^®^ flow cytometer (Thermo Fisher, Carlsbad, CA, USA), and analysis was performed using the Attune^®^ cytometric software v5.3.0. During the electronic data analysis, electronic gating was performed on the lymphocyte population using the forward scatter and side scatter properties. Within the lymphocyte subset, the NK cells were identified as CD3-negative CD56-positive cells, and the T lymphocytes identified based on CD3 expression. 

### 4.6. Immune Cell Recognition of Transformed Target Cells

The CD107a marker is constitutively expressed on the interior of lysosomes in cytotoxic cells. When NK cells [47] and cytotoxic T cells [48] engage in killing of transformed target cells, CD107a is transiently expressed on the cell surface [49,50], and the flow cytometric evaluation of the externalization of CD107a on cytotoxic cells is used as a tool to detect degranulation related to cytotoxic function [51]. The hematopoietic K562 cell line was obtained from the American Type Culture Collection (Manassas, VA, USA). Freshly purified human peripheral blood mononuclear cells (PBMC) re-suspended in RPMI 1640 were plated at 2 × 10^5^ cells/well in round-bottomed 96-well micro-assay plates and treated with serial dilutions of the test products in triplicate. Negative control wells in triplicate were left untreated. In addition, three wells containing PBMC alone and K562 cells alone served as negative controls for baseline CD107a expression, and one triplicate set of wells was treated with IL-2 (100 IU/mL) as a positive control. Target cells were K562 cells, an NK-cell sensitive tumor cell line widely used in NK cell cytotoxicity studies and were added at 2 × 10^4^ cells/well. The co-cultures of the two cell types were loosely pelleted by a brief 30-s centrifugation at 2400 rpm followed by incubation at 37 °C for 1 h. Monensin was added to block the cellular re-uptake of CD107a, and thus increase the detection on the cell surface. Cells were transferred to V-bottom microtiter plates for processing and staining. Cells were stained with CD3-PerCP, CD56-PE, and CD107a-FITC. The expression of CD107a on the NK cells was determined by flow cytometry. The CD3 negative, CD56 positive NK cells, and the CD3 positive T lymphocytes were differentiated from the K562 cells based on forward and side scatter properties, and from other lymphocytes by electronic gating on CD3 negative versus CD56, followed by evaluation of fluorescence intensity for CD107a. Samples were assayed in triplicate and experiments repeated three times using cells derived from three different blood donors.

### 4.7. Data Analysis

Averages and standard deviations for each data set were calculated using Microsoft Excel. Statistical analysis was performed using a 2-tailed independent *t*-test. Statistical significance was indicated when *p* < 0.05 and a high level of significance when *p* < 0.01.

## 5. Conclusions

Enhanced immune communication via cytokine production and defense activity towards target cells was enhanced when human immune cells were treated with very low doses of bovine colostrum peptides supportive of T cell cytotoxicity, in the presence of a complex blend of β-glucans from multiple plant-based and medicinal mushroom-based sources. This in vitro work warrants further work in vitro and in human clinical studies involving immune activation, immune surveillance, and eliciting changes in cytokine levels. 

## Figures and Tables

**Table 1:** Change in CD69 expression on immune cell subsets ^a^.

CD69 Expression (% Increase)	BC-Pep ^b^	IB ^c^	IB + BC-Pep ^d^	Significance ^e^
CD3− CD56+ NK cells	Not detectable	19.73 + 3.65	73.79 + 21.12	
CD3+ CD56+ NKT cells	17.20 ± 37.00	16.94 ± 11.90	111.55 ± 2.16	* ##
CD3+ CD56− T cells	1.61 ± 1.38	4.86 ± 1.70	18.68 ± 4.94	* #

^a^ Dose of the immune blend (IB) was 1 mg/mL and the dose of bovine colostrum peptides (BC-Pep) was 16 µg/mL to match the content of BC-Pep factors in IB + BC-Pep. ^b^ BC-Pep: Bovine colostrum low-molecular weight peptides. ^c^ IB: Immune blend containing botanical and fungal non-starch polysaccharides. ^d^ IB + BC-Pep: Both IB and BC-Pep were added to the immune cell cultures. ^e^ Statistical indicators: (*): *p*-value below 0.1 when compared to BC-Pep alone, *: *p*-value below 0.05 when compared to BC-Pep alone, #: *p*-value below 0.05 when compared to IB alone, ##: *p*-value below 0.01 when compared to IB alone.

## Data Availability

The data presented in this study are available on request from the corresponding author upon reasonable request.
